# Results of Modified Minimally Invasive Hallux Valgus Surgery, Factors for the First Metatarsal Shortening, and Patients’ Satisfaction

**DOI:** 10.3390/jcm13247840

**Published:** 2024-12-22

**Authors:** Jihyun Hwang, Jung-Ah Cho, Go Woon Choi, Si Young Song, Collin Lee, Sung Jae Kim

**Affiliations:** 1 Department of Biomedical Engineering, Johns Hopkins University School of Medicine, Baltimore, MD 21205, USA; jhwang50@jh.edu; 2 Department of Orthopedic Surgery, Hallym University Dongtan Sacred Heart Hospital, Hwaseong 18450, Republic of Korea; jungahcho@dgist.ac.kr (J.-A.C.); goni8586@gmail.com (G.W.C.); superdsys@hotmail.com (S.Y.S.); 3 Daegu Gyeongbuk Institute of Science and Technology, School of Undergraduate Studies, College of Transdisciplinary Studies, Daegu 42988, Republic of Korea; 4 Department of Biology, University of Maryland, College Park, MD 20742, USA; clee1241@terpmail.umd.edu

**Keywords:** hallux valgus, bunion, minimally invasive, MIS, technique, osteotomy

## Abstract

**Background**: Hallux valgus is a prevalent foot deformity conventionally treated with open surgical techniques, which carry risk of complications due to extensive soft tissue dissection. Minimally invasive surgeries (MISs) as alternatives offer comparable outcomes, reduced pain, and faster recovery; however, their challenges include the risk of shortening of the first metatarsal. This study aimed to assess the efficacy of our modified MIS hallux valgus correction technique and investigate the factors that affect first metatarsal shortening. **Methods**: Twenty-nine feet treated with modified MIS hallux valgus surgery between 2017 and 2022 were included with an average follow-up of 29 months. Clinical outcomes were evaluated with the Manchester–Oxford Foot Questionnaire (MOXFQ), Foot Function Index (FFI), and visual analog scale for pain. Radiographic evaluations included the intermetatarsal angle (IMA), hallux valgus angle (HVA), distal metatarsal articular angle (DMAA), first metatarsal length, and sesamoid grade. **Results**: Functional and radiographic outcomes were significantly improved (*p* < 0.0001 and *p* < 0.001, respectively). Significant correlations between patient satisfaction and the MOXFQ, FFI, and VAS scores were found, with no significant correlations between patient satisfaction and radiographic outcomes. Non-purchasing of the lateral cortex of the proximal osteotomy site was identified to increase risk of first metatarsal shortening (odds ratio [OR] = 22.09, *p* = 0.0064). **Conclusions**: Our modified MIS for hallux valgus correction showed favorable radiographic and functional outcomes. Proximal lateral cortex purchasing should be targeted to reduce postoperative shortening of the first metatarsal.

## 1. Introduction

Hallux valgus is a prevalent foot deformity characterized by a lateral deviation of the great toe and medial deviation of the first metatarsal, which can lead to pain, functional impairment, and footwear difficulties [1,2]. Conventional surgical techniques, such as the scarf or Chevron osteotomy, are known to be effective in correcting deformity but involve extensive soft tissue dissection, possibly increasing the risks of complications such as wound infections and delayed bone healing [3,4,5]. In contrast, minimally invasive surgery (MIS) techniques aim to achieve similar clinical and radiographic outcomes with smaller incisions, reduced soft tissue disruption, and potentially fewer complications [6,7]. MIS for hallux valgus correction has gained increasing interest in the orthopedic field due to its potential benefits over traditional open surgical techniques and possible patient satisfaction due to its cosmetic advantages of small incisions.

Advances in imaging techniques, specialized surgical instruments, and the development of novel osteotomy techniques have facilitated refining MIS procedures [8,9]. Clinical studies have reported that minimally invasive procedures, such as percutaneous Chevron–Akin osteotomies, can provide comparable results to traditional techniques while reducing postoperative pain and accelerating recovery [10,11]. For instance, a matched cohort study reported that the third-generation minimally invasive Chevron–Akin osteotomy achieved similar clinical and radiological outcomes as the conventional scarf–Akin osteotomy over a two-year follow-up period [6].

Despite these promising results, adopting MIS for hallux valgus correction still has some burdens. The learning curve for surgeons for MIS techniques seems to be very steep, requiring specialized training and experience to ensure the accuracy of techniques and avoid complications [12,13]. In addition, concerns exist regarding the long-term durability of MIS procedures, particularly in patients with severe deformities or associated foot conditions [14,15]. Comparative studies have demonstrated that while MIS techniques can achieve comparable early outcomes to traditional surgical techniques, further research is needed to confirm their efficacy over long-term follow-up periods [16].

One of the concerns for MIS procedures is the risk of the first metatarsal shortening, which can result from the nature of the specific osteotomy techniques used. Shortening of the first metatarsal is a known risk in some minimally invasive approaches, particularly those involving percutaneous distal osteotomies. Studies have indicated that the Bosch or Reverdin-Isham procedures can lead to significant shortening and complications like transfer metatarsalgia, which may impact clinical outcomes and patient satisfaction [17,18]. Meta-analyses comparing MIS and open techniques have generally shown no significant differences in first metatarsal shortening; however, individual reports highlight the importance of precise techniques to minimize this risk [19,20]. The conventional tool for MIS, the Shannon burr, has a thickness of 2 mm. Due to the inherent thickness of the burr itself, shortening is more likely to occur with the current technique. Shortening of the hallux can disrupt the normal parabola of the foot, potentially causing transfer metatarsalgia to the lesser toes. This may weaken toe propulsion during the normal gait cycle, thereby negatively impacting patient satisfaction.

We have performed MIS hallux valgus surgery with some modifications that use pre-2.0 mm Kirschner wiring for optimal screw positioning and that use a 1.1mm width Midas burr for less shortening with reduced width of the burr head. The current study aims to introduce some technical tips for reproducible MIS hallux valgus surgery and evaluate the surgical outcome of our modified MIS hallux valgus correction surgery.

## 2. Materials and Methods

### 2.1. Study Design

This study was a retrospective observational single-surgeon case series of consecutive patients. Ethical approval for this study was obtained from the IRB/EC of our institute (HDT 2024-11-007), and all patients were permitted to provide informed consent. During the period between January 2017 and June 2022, patients who underwent primary correction of hallux valgus deformity in our institution were enrolled in the current study. Indications of surgery were clinically made based on symptoms of the deformity. Symptoms included pain or discomfort in the bunion area in footwear, transfer metatarsalgia, and symptomatic lesser-toe deformities related to hallux valgus deformity. A relative contraindication for MIS in hallux valgus correction was elderly osteoporotic patients, due to concerns regarding fixation failure associated with small bone contact areas. Inclusion criteria were patients who received an MIS osteotomy procedure for surgical correction of the hallux valgus deformity and who were followed for a minimum of two years. Patients who underwent additional forefoot procedures, like distal metaphyseal metatarsal osteotomy for lesser toes, were also included for radiographic outcome evaluation. Exclusion criteria were those who had previous hallux valgus surgery or who had a degenerative first metatarsophalangeal joint, patients who missed their clinical evaluation survey, patients who were lost to follow-up, and those who underwent surgical correction with other traditional open osteotomies, like distal or proximal Chevron osteotomy.

### 2.2. Clinical Outcome Evaluation

We routinely complete the Manchester–Oxford Foot Questionnaire (MOXFQ) and Foot Function Index (FFI) for clinical evaluation preoperatively, and they were also completed for each patient at final follow-up. The MOXFQ consists of 3 domains (walking and standing, social interaction [based on the subjective patient’s consciousness regarding their feet, as well as the overall impact on social, work, and other daily activities], and pain), with the score in each domain ranging from 0 (best possible) to 100 (worst possible) points. An overall summary “index” score was also calculated [21]. The minimal clinically important difference (MCID) of the MOXFQ has been evaluated to be 16, 12, and 24 for the walking and standing, pain, and social interaction domains, respectively [22]. The Foot Function Index (FFI) was also used to assess the functional status and pain of participants related to foot conditions. The FFI is a self-reported subjective questionnaire developed to evaluate the impact of foot conditions on function, measuring pain, disability, and activity limitations [23]. The tool has undergone translation, cultural adaptation, and validation in various languages, demonstrating reliability and validity across multiple populations [24]. The FFI consists of 23 items divided into three subscales: pain (9 items), disability (9 items), and activity limitation (5 items). Participants rated their experiences over the past week on a Likert scale from 0 (no pain or difficulty) to 10 (worst pain or extreme difficulty). Each subscale score was calculated by averaging the respective item scores, and the overall FFI score was calculated by averaging the scores of the three subscales. Higher scores indicate greater impairment of foot function. Participants completed the FFI questionnaire at baseline and post intervention to evaluate changes in foot function over time. The reliability and validity of the FFI have been supported in various studies, including a validated Korean version showing high reliability and validity for use with Korean populations [25].

The visual analog scale (VAS) was evaluated for overall pain in the foot at last follow-up. Patient satisfaction was evaluated via a 5-tiered survey, with the choices of very satisfied, satisfied, moderate, disappointed, or very disappointed. Postoperative complications were identified during follow-up.

### 2.3. Radiographic Outcome Evaluation

For radiographic outcome evaluation, weight-bearing radiographs were taken preoperatively, at 6 weeks postoperative, and at final follow-up. The following radiographic parameters were measured for all patients according to the American Orthopaedic Foot & Ankle Society technique and were categorized according to deformity severity [26].

The 1–2 intermetatarsal angle (IMA), hallux valgus angle (HVA), distal metatarsal articular angle (DMAA), 1st metatarsal length, and sesamoid grade were measured on anteroposterior weight-bearing radiograph. The Meary angle was also measured to evaluate the lateral weight-bearing radiograph to check accompanying flat foot deformity. Screw purchasing of the lateral cortex proximal osteotomy bone was also evaluated. Another independent orthopedic surgeon repeated all radiographic measures, and intra-rater reliability was calculated.

### 2.4. Statistical Analysis

A paired *t*-test or a Wilcoxon signed-rank test was used to compare surgical outcomes according to the parametricity of data. Normality was tested via the Shapiro–Wilk test (*n* = 28). Univariate and multivariate regression analyses were also performed to evaluate factors affecting postoperative 1st MT shortening, and separate regression analysis was also performed to assess factors affecting the final MOXFQ. Significance was defined as *p* < 0.05. All analyses were performed with SPSS version 18.0 (IBM Corp., Armonk, NY, USA).

### 2.5. Surgical Technique

The procedure is performed under popliteal and femoral nerve blocks without the use of a tourniquet. This approach allows natural bleeding during the surgery, which helps to prevent thermal burns during the burring process. Adequate draping is essential, ensuring the drape extends well above the thigh, allowing free movement of the knee joint. The ipsilateral hip is elevated using surgical towels to position the ankle in a neutral position naturally. The assistant flexes the knee to a position where the foot anteroposterior view can be easily observed using portable fluoroscopy during a surgical procedure.

**Figure 1:**
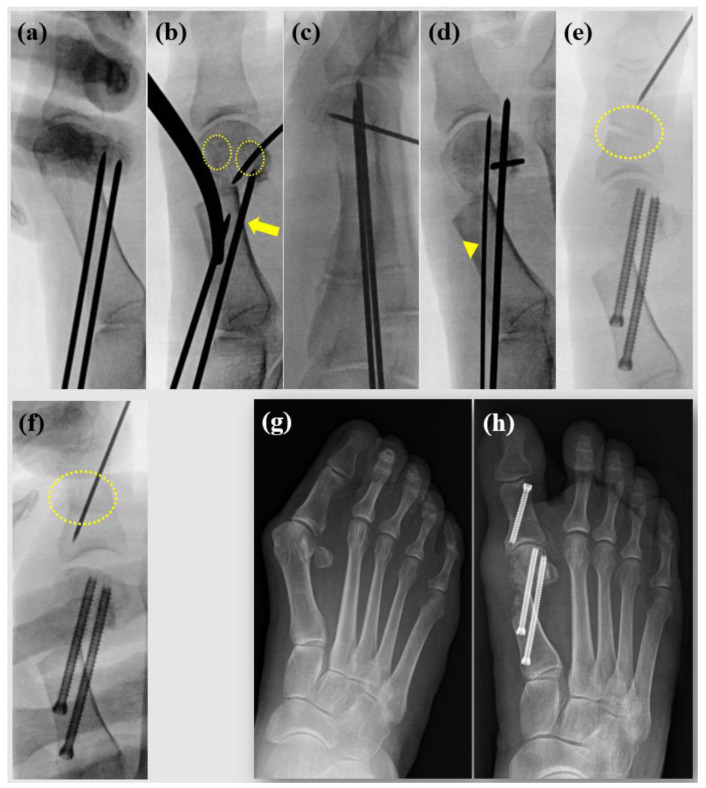
Surgical procedures. (**a**) Pre K-wiring for ideal screw positioning before osteotomy. (**b**) Sesamoid reduction with metatarsal head rotation (dotted circles), purchasing lateral cortex of proximal metatarsal (arrow). (**c**) Check lateral image to confirm proper sagittal alignment. (**d**) K-wires are exchanged manually to guide wire for screw insertion (arrowhead). (**e**) During Akin osteotomy procedure, ensuring that the lateral cortex is preserved and is advantageous for precise medial closing, (**f**) ensure that the medial closing is precisely achieved before inserting the guide pin. (**g**) Preoperative radiograph. (**h**) Postoperative radiograph.

First, a 2.0 mm Kirschner wire (K-wire) is used to predefine the trajectory for fixation screws. This step ensures an optimal direction for screw insertion after the osteotomy (Figure 1a). The pre-inserted K-wires are then partially withdrawn proximally beyond the planned osteotomy site. A small incision (~3 mm) is made just proximal to the sesamoid complex. To prevent 1st metatarsal shortening caused by the burr thickness and lateral shift of the 1st metatarsal, a perpendicular osteotomy is performed on the 1st metatarsal using a Midas Rex^®^ Legend^®^ burr (Medtronic, Dublin, Ireland). The burr speed is set to 8000 rpm, and constant irrigation using a syringe is maintained to prevent thermal burns. Once the osteotomy is complete, a large Kelly forceps is used to translate the distal osteotomy fragment laterally. A 1.6 mm K-wire is inserted into the metatarsal head to stabilize the fragment, which is supinated to correct pronation deformity and achieve sesamoid reduction (dotted circles, Figure 1b). It is ensured that the proximal K-wire securely purchases the lateral cortex of the proximal segment (arrow). If a DMAA correction is required, the K-wire may be adjusted and used as a joystick to fine-tune alignment. Once the distal fragment is translated to the desired position, the pre-inserted 2.0 mm K-wire is reinserted to fix the osteotomy temporarily. In the figure-of—4 position, the foot’s lateral view is checked to confirm the absence of sagittal plane deformities (Figure 1c). After verifying the proper positioning and fixation of the distal fragment and K-wires, the distal 2.0 mm K-wire is replaced with a guide wire manually for screw insertion (Figure 1d). Drilling is performed, and a cannulated screw system (Jeil Medical Corporation, Seoul, Korea) is used to fixation. The same technique is applied for the proximal screw fixation, completing the stabilization process. If MTP joint incongruency is observed, a beaver knife is used to release the adductor tendon, lateral MTP joint capsule, and lateral metatarso-sesamoid ligament. The hallux is gently overcorrected to ensure adequate release of the lateral joint space. If necessary, an Akin osteotomy is performed using the Midas burr. Care is taken to preserve enough of a portion of the lateral cortex of the proximal phalanx. Fluoroscopy confirms a precise medial closing wedge osteotomy before inserting guide wire for screw fixation (dotted circles, Figure 1e,f). Pre- and postoperative radiographs are shown (Figure 1g,h).

### 2.6. Postoperative Care

**Figure 2:**
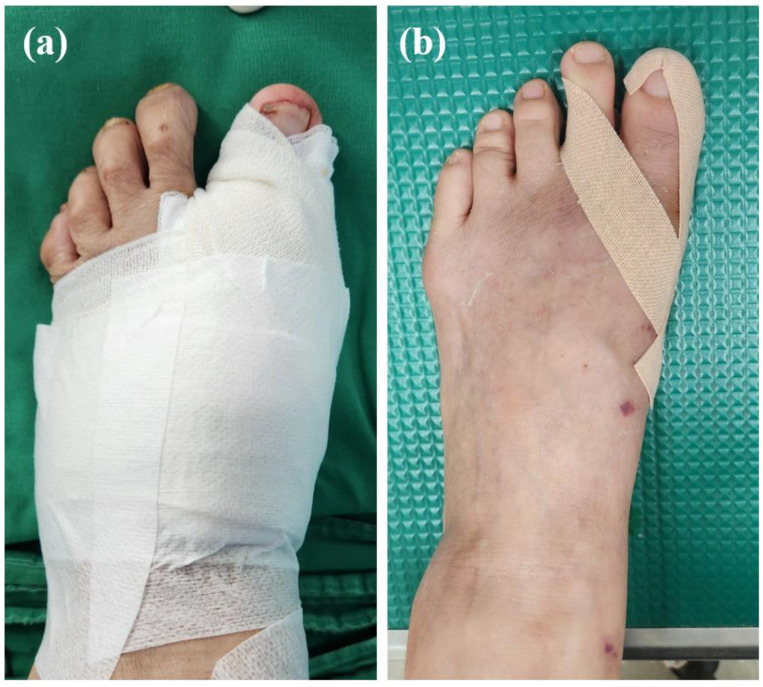
Postoperative protocol. (**a**) A 2 inch elastic bandage was applied with the hallux in a slightly overcorrected position during the first 6 weeks following surgery; (**b**) after 6 weeks, kinesio tape was applied for 1–2 more months.

**Figure 3:**
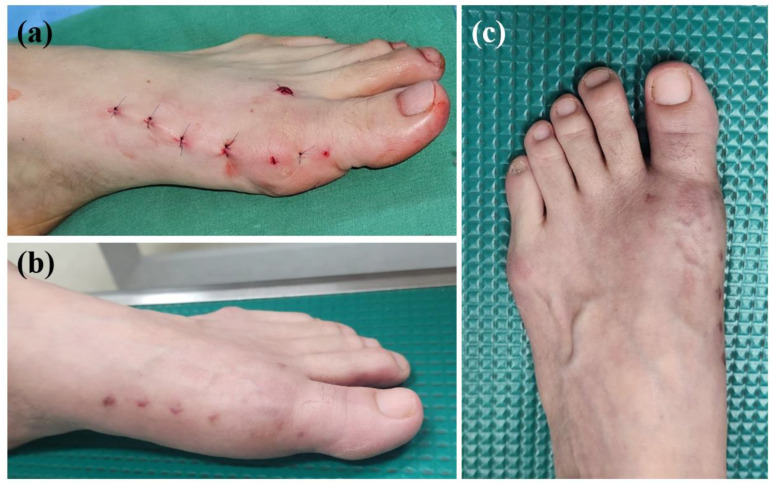
(**a**) Postoperative wound; (**b**) surgical wound two months after surgery; (**c**) surgical wound three months after surgery.

Patients are permitted to walk with hard-sole postoperative shoes on day one after surgery. Gentle range of motion exercises of the hallux are encouraged two weeks following surgery. Because the MIS surgical procedure for hallux valgus correction does not regularly perform medial capsulorrhaphy, careful bandaging to keep the hallux in a slightly overcorrected position during the first six weeks following surgery is important (Figure 2). After 6 weeks, the elastic bandage is changed to kinesio taping until patients tolerate it, at least one more month. Usually, 3 months following surgery, an excellent aesthetical postoperative wound can be expected (Figure 3).

## 3. Results

**Figure 4:**
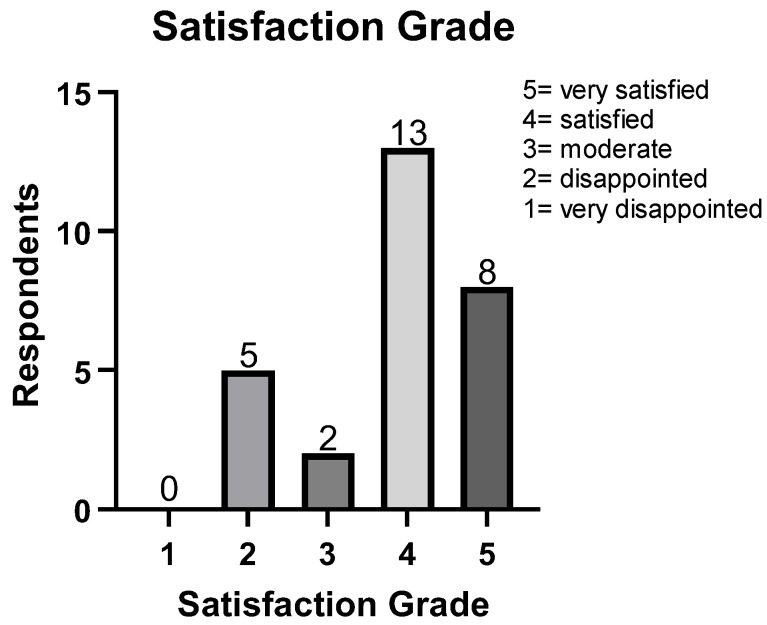
Patients’ satisfaction at final follow-up.

The demographics of the current study subjects are summarized in Table 1. Functional outcome evaluation results are listed in Table 2. All functional outcome measures were significantly improved after surgery. However, for patients’ satisfaction analysis, 5 out of the total 29 patients (17.2%) were disappointed with the surgical results (Figure 4). Details of the patients with disappointing surgical results are listed in Table 3. One patient recurred deformity of HVA with 24.6° (preoperatively 36.6°) and IMA of 8.6° (preoperatively 14.0°). Other patients with disappointing surgical results showed favorable HVA and IMA at final follow-up.

**Figure 5:**
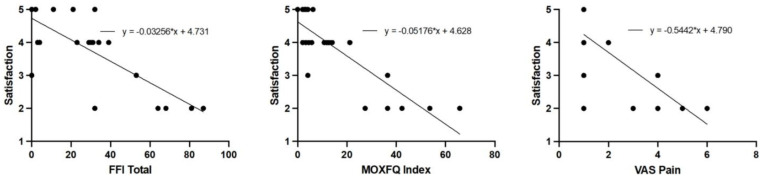
Correlation curve for patients’ satisfaction. Only functional outcomes (FFI, MOXFQ, and VAS for pain) showed significant correlations with patients’ satisfaction.

Radiographic outcomes are summarized in Table 4. All the measurement values showed acceptable measurement reliability (intraclass correlation coefficients = 0.765 to 0.923). All the radiographic indexes were significantly improved at the final follow-up. Regression analysis results for factors affecting secondary first metatarsal shortening are summarized in Table 5. Un-purchasing the lateral cortex of the proximal osteotomy site showed a significantly high odds ratio (odds ratio [OR] = 22.09, 95% confidence interval [CI] = 3.05 to 320.40, *p* = 0.0064). The correlation analysis for patients’ satisfaction is summarized in Table 6 and Figure 5. Only the functional outcome measures showed a significant correlation with patients’ satisfaction. The MOXFQ showed the highest correlation (Spearman’s rho = −0.80, 95% CI = −0.91 to −0.60, *p* < 0.001).

**Figure 6:**
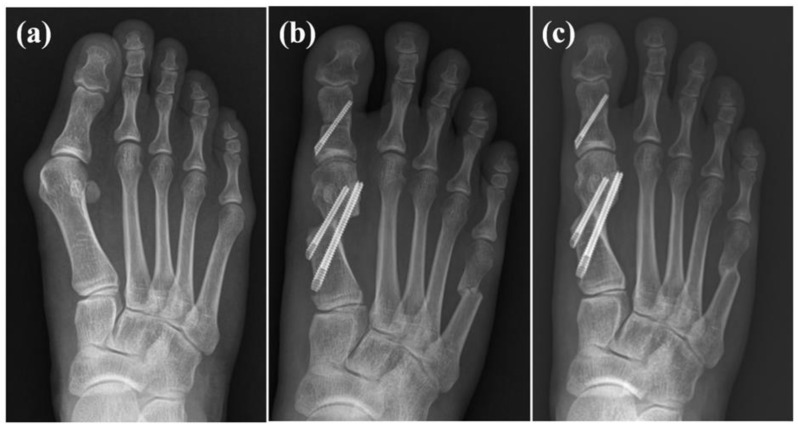
Case example of female, 35-year-old patient. (**a**) Preoperative radiographs show moderate hallux valgus deformity; (**b**) 6 weeks following surgery, bunionette correction was also performed simultaneously; (**c**) 2 years following surgery.

Complications were present in one patient who showed screw pull-out during follow-up, with favorable functional and radiographic outcomes. Two patients presented recurrent hallux valgus: one patient reported satisfactory surgical results along with significantly improved pain scores, while the other reported disappointing results. An example case is depicted in Figure 6.

## 4. Discussion

The main finding of the current study is that our modified minimally invasive surgery (MIS) for hallux valgus correction provides favorable clinical and radiological results. Our findings indicate significant improvements in both functional and radiographic parameters, with high patient satisfaction rates. Postoperative pain was markedly reduced, and radiological foot alignment was significantly restored to within the normal range. Factors affecting secondary first MT shortening seem to be the screw un-purchasing of proximal lateral cortex. Patients’ satisfaction seems to be not significantly related to radiographic results, but significantly related to functional outcomes.

MIS for hallux valgus has been extensively studied, with numerous reports describing favorable outcomes. Brogan et al. reported that their 49 cases of MIS hallux valgus provide substantial improvements in pain relief and functional outcomes in mild to moderate hallux valgus cases, comparable to traditional open distal Chevron osteotomy [27]. Kaufmann et al., in a randomized controlled trial, found that MIS yields comparable correction angles to open techniques while reducing soft tissue trauma and patient discomfort [28]. Ji et al.’s meta-analysis corroborated these findings, showing that MIS provides comparable correction to open surgery with reduced early postoperative discomfort [19]. Our findings corroborate previous results of MIS hallux valgus surgery in functional and radiological outcomes.

However, MIS for hallux valgus also presents certain limitations. A steep learning curve for surgeons can increase complication rates [12,29]. Reliable fixation of the distal osteotomy fragment also poses significant challenges in MIS procedures, and it may contribute to the risk of postoperative displacement or delayed union, which requires careful intraoperative technique to mitigate [30]. In addition, some studies noted that the limitation of surgical field visualization in MIS may restrict its application in more complex deformities, potentially limiting its use to mild to moderate cases [31,32]. These factors emphasize the importance of sufficient training and careful patient selection to optimize outcomes with MIS.

A specific concern in MIS hallux valgus correction is the shortening of the first metatarsal, which can have biomechanical consequences. Geng et al. examined this effect and suggested that shortening up to 6 mm is within a safe range, but excessive shortening increases the load on the central metatarsals, raising the risk of transfer metatarsalgia [33]. Jung et al. further explored the biomechanical impact, showing that even slight metatarsal shortening can significantly alter plantar pressure distribution, potentially causing forefoot pain [20]. These findings indicate that controlled first metatarsal shortening is critical to avoid postoperative complications in MIS hallux valgus surgery.

The current study analyzed the risk factors related to first MT shortening during the follow-up period. We revealed that screw un-purchasing of the proximal lateral cortex showed a significantly high odds ratio for metatarsal shortening after surgery. The penetration of the proximal lateral cortical bone by the screw is crucial for the stability of fixation. If this is not achieved, the screw may move within the cancellous bone during the weight-bearing process, leading to instability and potential misalignment.

The current study has some limitations. First, it is a retrospective observational study with a small number of cases. However, we also performed a clinically meaningful analysis of risk factors for first MT shortening and factors related to patient satisfaction. Second, for more clinically relevant, meaningful research, a control group should be used to compare the results of MIS techniques to traditional ones, like distal Chevron osteotomy. Third, we included some patients with additional lesser toe weil osteotomy procedures. However, weil osteotomy seems not to affect the radiographic outcomes of HVA, IMA. DMAA, and first MT length, but it may affect functional outcomes. Although the functional impact of weil osteotomy on hallux valgus correction may be minimal, caution is warranted in interpreting the results of the current study.

## 5. Conclusions

In conclusion, our modified MIS hallux valgus correction surgery using Midas burr showed favorable radiographic and functional results. Proximal lateral cortex screw purchasing should be targeted to reduce postoperative first metatarsal shortening.

## Figures and Tables

**Table 1:** Demographic data of patients in the current study.

Demographic Data	
Age (Mean, SD)	55.54 ± 11.89
Sex (male/female)	1:27
Mean follow-up (months)	29.3 ± 4.26
HV severity grade	
Mild	10
Moderate	17
Severe	1

SD, standard deviation; HV, hallux valgus.

**Table 2:** Functional outcome measures following MIS hallux valgus correction.

Outcome Measure	Preoperative		Last Follow-Up		Changes		*p*
	Mean ± SD	Median (IQR)	Mean ± SD	Median (IQR)	Mean ± SD	Median (IQR)	
MOXFQ Index	52.29 ± 11.06	51.67 (46.32–57.02)	14.89 ± 17.15	8.503 (3.33–19.38)	37.41 ± 15.22	41.74 (24.20–50.45)	<0.0001
Walking and Standing	49.85 ± 12.23	46.41 (42.84–56.23)	11.22 ± 14.47	3.570 (0–28.56)	38.63 ± 13.64	39.27 (28.56–49.09)	<0.0001
Pain	46.11 ± 16.37	40.00 (40.00–55.00)	15.18 ± 21.32	10.00 (1.25–10.00)	29.29 ± 20.89	30.00 (15.00–43.75)	<0.0001
Social	62.72 ± 11.72	62.50 (56.25–68.75)	18.30 ± 19.46	15.63 (0.00–25.00)	44.42 ± 19.93	46.88 (26.56–60.94)	<0.0001
FFI Total	80.36 ± 14.42	80.50 (68.25–90.00)	26.82 ± 24.78	23.00 (3.25–33.50)	53.54 ± 23.64	53.50 (32.00–73.00)	<0.0001
FFI (Pain)	38.29 ± 7.21	41.00 (31.25–42.00)	13.39 ± 11.90	19.50 (10.00–41.00)	24.89 ± 12.10	25.50 (14.25–35.50)	<0.0001
FFI (Disability)	42.07 ± 7.50	40.50 (36.50–58.00)	13.43 ± 13.32	13.00 (2.0–16.76)	28.64 ± 12.05	27.00 (18.25–37.75)	<0.0001
VAS for Pain	4.536 ± 1.07	4.5 (4.0–5.0)	1.714 ± 1.384	1.0 (1.0–2.0)	2.821 ± 1.09	3.0 (2.0–4.0)	<0.0001

MOXFQ, Manchester–Oxford Foot Questionnaire; FFI, Foot Function Index; VAS, visual analog scale; SD, standard deviation; IQR, interquartile range.

**Table 3:** Summary of patients’ details with disappointing surgical results.

	Sex	Age	Pre HVA	Final HVA	Pre IMA	Final IMA	Final Sesamoid Grade	MT Length Change	MOXFQ	FFI	VAS
#1	F	60	20.6	1.1	11.2	0.6	2	–9.4	27.4	32	1
#2	F *	75	26.8	1.0	12.9	6.1	4	–3.0	65.7	87	6
#3	F *	75	26.6	–2.1	12.7	8.8	4	–4.2	53.6	81	5
#4	F	58	36.6	24.6	14	8.6	4	–2.8	42.3	68	3
#5	F	56	24.8	7.1	13.7	3.5	2	–8.1	36.5	64	4

* Second and third row are the same patient with both feet.

**Table 4:** Radiographic outcomes of MIS hallux valgus correction surgery.

	Preoperative	6th Week	Final	*p*	*p* ^2^
IMA	12.98 ± 2.67	4.889 ± 3.59	5.83 ± 2.93	<0.0001	0.164
HVA	26.60 (21.65–33.10)	4.450 (1.63–7.10)	6.20 (1.80–9.28)	<0.0001	0.544
DMAA	7.35 (5.0–11.10)	-	4.350 (6.30–0.40)	0.0005	-
Sesamoid grade	5.0 (4.0–6.0)	-	2.5 (2.0–4.0)	<0.0001	-

Data are given as mean ± standard deviation or median (interquartile range) according to the normality of the data set; *p*, preop vs. final; *p*^2^, 6th week vs. final, statistical significance set to *p* values of 0.025 here (= 0.05/2) for multiple analyses with the same data set (Bonferroni correction). IMA, intermetatarsal angle; HVA, hallux valgus angle; DMAA, distal metatarsal articular angle; sesamoid grade (range 1 to 7).

**Table 5:** Factors that affect the 1st metatarsal shortening.

	Odds Ratio (95% C.I.)	*p*
Age	1.040 (0.9376, 1.185)	0.4901
No cortex purchasing	22.09 (3.047, 320.4)	0.0064
Meary angle	0.8993 (0.7445, 1.039)	0.1894

More than 6 mm of shortening defined as metatarsal shortening; C.I., confidence interval.

**Table 6:** Correlation analyses for variable with patients’ satisfaction.

Correlation	FFI Total	MOXFQ Index	VAS Pain
ρ (Spearman)	−0.68	−0.80	−0.63
95% CI	−0.84 to −0.40	−0.91 to −0.60	−0.82 to −0.33
*p*	<0.001	<0.001	0.0003

Other variables did not show significant correlations with patients’ satisfaction.

## Data Availability

The data used/generated for the current study are not publicly available due to private information but are available from the corresponding author on reasonable request.
